# XRCC1 serves as a potential prognostic indicator for clear cell renal cell carcinoma and inhibits its invasion and metastasis through suppressing MMP-2 and MMP-9

**DOI:** 10.18632/oncotarget.22680

**Published:** 2017-11-25

**Authors:** Qing-Hua Liu, You Wang, Hong-Mei Yong, Ping-Fu Hou, Jie Pan, Jin Bai, Jun-Nian Zheng

**Affiliations:** ^1^ Jiangsu Key Laboratory of Biological Cancer Therapy, Xuzhou Medical University, Xuzhou 221002, Jiangsu Province, China; ^2^ Department of Pathology, Xuzhou Medical University, Xuzhou 221002, Jiangsu Province, China; ^3^ Department of Obstetrics and Gynecology, Renji Hospital, School of Medicine, Shanghai Jiao Tong University, Shanghai 200127, China; ^4^ Department of Medical Oncology, Huai’an Hospital to Xuzhou Medical University, Huai’an 223001, Jiangsu Province, China; ^5^ Jiangsu Center for The Collaboration and Innovation of Cancer Biotherapy, Cancer Institute, Xuzhou Medical University, Xuzhou 221002, Jiangsu Province, China; ^6^ Center of Clinical Oncology, Affiliated Hospital of Xuzhou Medical University, Xuzhou 221002, Jiangsu Province, China; ^7^ Department of Clinical Oncology, Pizhou people's Hospital, Xuzhou 221002, Jiangsu Province, China

**Keywords:** XRCC1, renal cell carcinoma, metastasis, prognostic biomarker, MMPs

## Abstract

X-ray repair cross-complementing group 1 (XRCC1) is a major DNA repair gene that is responsible for fixing DNA base damage and single-strand breaks by interacting with DNA components at the damage site. This study explored the clinical significance of XRCC1 in human clear cell renal cell carcinoma (ccRCC) and further examined the mechanism of the role of XRCC1 in ccRCC. The clinical relevance of XRCC1 in ccRCC was evaluated using tissue microarrays and immunohistochemical staining of two independent human ccRCC cohorts. Our data demonstrated that XRCC1 expression was dramatically decreased in ccRCC tissues compared with that in normal renal tissues and paired adjacent non-tumor tissues. Low XRCC1 expression was significantly correlated with lymph node metastasis and with worse overall and disease-specific survival in patients, as determined by log-rank tests. However, Cox regression analysis revealed that XRCC1 expression was not an independent prognostic factor in ccRCC patients. Furthermore, XRCC1 suppressed ccRCC migration and invasion by inhibiting MMP-2 and MMP-9 expression through the regulation of TIMP-2 and TIMP-1. These findings indicated that decreased XRCC1 expression was associated with lymph node metastasis but was not an independent prognostic factor in ccRCC patients. XRCC1 may serve as a potential therapeutic target for inhibiting ccRCC metastasis but cannot be used as an independent prognostic factor.

## INTRODUCTION

The most common urological tumor in adults is renal cell carcinoma (RCC), which accounts for approximately 3% of human malignancies worldwide [1]. Clear cell renal cell carcinoma (ccRCC) accounts for 85%∼90% of all RCC cases and has the worst prognosis of any RCC type, due to local recurrence and distant metastasis [2]. Approximately half of ccRCC patients develop metastatic diseases, which are usually incurable, and the median survival of metastatic ccRCC patients is significantly worse than that of non-metastatic ccRCC patients [3, 4]. Therefore, it is very important to discover new effective biomarkers associated with ccRCC metastasis.

DNA repair plays a vital role in maintaining genetic integrity, and deficiencies in DNA damage repair enzymes, which are connected to many different types of diseases, specifically increase a person’s risk for developing cancer. XRCC1 (X-ray repair cross-complementing group 1) is a DNA repair gene that belongs to the XRCC family [5]. Many studies have investigated the association between XRCC protein expression and their roles in variant cancers. However, the role of XRCC1 in tumors is contradictory; low expression levels of XRCC1 were observed in gastric and pancreatic cancer and were related to invasiveness [6, 7], but a high expression of XRCC1 was observed in head and neck squamous cell carcinoma and was related to a worse patient prognosis [8]. In addition, little is known about the role of XRCC1 in ccRCC.

We aimed to determine the roles of XRCC1 protein in ccRCC patients in this study. We investigated the expression of XRCC1 and the clinicopathological and prognostic significance of XRCC1 in ccRCC. Our results showed that low XRCC1 expression was significantly associated with ccRCC progression. Meanwhile, we further investigated the mechanism by which XRCC1 regulates the invasion and metastasis of ccRCC through the TIMP/MMP pathway. Our results indicated that XRCC1 is a potential prognostic marker and therapeutic target for ccRCC.

## RESULTS

### The expression of XRCC1 is reduced in ccRCC compared with non-tumor tissues

**Figure 1:**
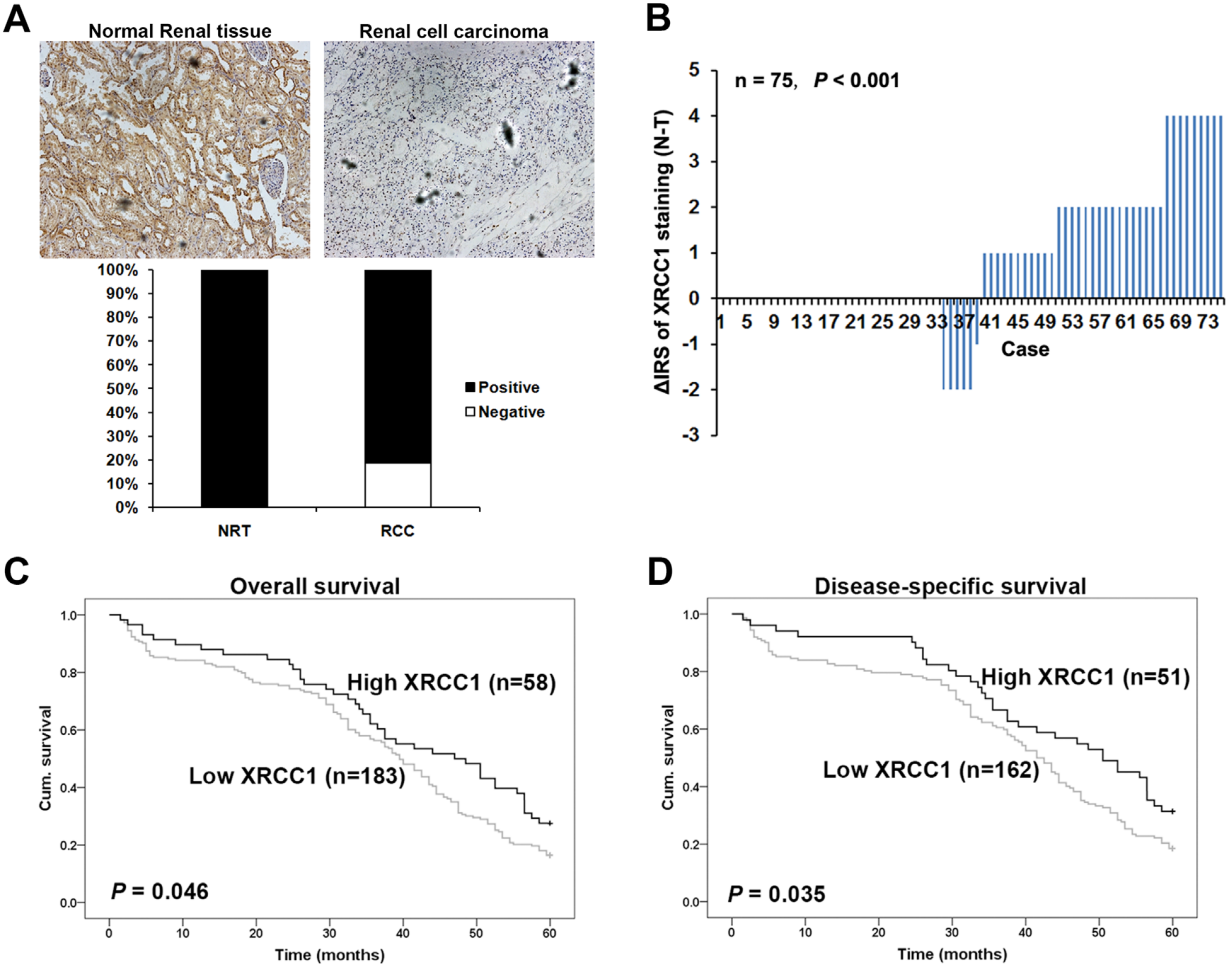
XRCC1 is decreased in ccRCC and associated with worse 5-year overall and disease-specific survival in ccRCC patients **(A)** Top panel, XRCC1 immunohistochemical staining in ccRCC and normal renal tissues, ×400. Bottom panel, XRCC1 was lower in ccRCC than in normal renal tissues. Immunohistochemical staining data was available from the validation cohort which including 35 normal renal tissues and 300 ccRCC. **(B)** Distributions of the difference in XRCC1 staining (ΔIRS=IRS_N_-IRS_T_). Immunoreactivity scores (IRS) of XRCC1 staining were available from the training cohort which including 75 pairs of tissues. The expression of XRCC1 was lower in tumor tissues (T) than the paired adjacent non-tumor tissues (N). **(C)** Low expression of XRCC1 correlated with worse 5-year overall cumulative survival of 241 ccRCC patients (*P*=0.046, log-rank test). **(D)** Low expression of XRCC1 correlated with worse 5-year disease-specific cumulative survival for 213 ccRCC patients (*P*= 0.035, log-rank test). Cum. Indicates cumulative.

We performed immunohistochemistry with the TMAs to study whether XRCC1 expression is changed in human ccRCC. Immunohistochemical staining was used with TMA slides to evaluate XRCC1 expression in ccRCC and paired adjacent non-tumor tissues. Samples with an IRS of 0-3 were classified as low-expressing, and samples with an IRS of 4-12 were identified as having high XRCC1 expression. In the validation TMA cohort containing 300 ccRCC cases and 35 cases normal renal tissues (Figure 1A, top panel), negative XRCC1 expression was observed in approximately 20% of the ccRCC samples but in none of the normal renal tissues (*P* <0.001, Figure 1A bottom panel). These results were in accordance with the other renal TMA including 75 pairs of ccRCC specimens and paired adjacent renal tissues. We found that the expression of XRCC1 was significantly lower in the ccRCC samples than in the paired normal tissues (*P* <0.001, Figure 1B). Taken together, the expression of XRCC1 expression was reduced in ccRCC tissues compared the expression in non-tumor tissues.

### Decreased XRCC1 expression correlates with clinicopathological parameters in ccRCC patients

The clinicopathological characteristics of the training cohort and the validation cohort of ccRCC biopsies are summarized in Table 1. As shown in Table 1, two-tailed Fisher’s exact analysis revealed that XRCC1 expression in the carcinoma tissues of the training cohort clearly correlated with lymph node metastasis pN status (*P*=0.031). This finding was confirmed in the validation cohort of ccRCC patients (Table 1). However, we did not find a significant correlation between PinX1 expression and other clinicopathologic features, including age, gender, tumor size, depth of invasion-pT status and TNM stage, in either the training cohort or the validation cohort.

**Table 1:** Relationship between XRCC1 staining and clinicopathological characteristics of the individuals in two cohorts of ccRCC patients

Variables	Training cohort (75 cases)			Validation cohort (300 cases)		
	Low (%)	High (%)	*P* ^*^	Low (%)	High (%)	*P* ^*^
**Age**						
≤56 years	17 (47.2)	19 (52.8)	0.216	105 (45.3)	38 (26.6)	1.000
>56 years	10 (41.0)	23 (59.0)		116 (73.9)	41 (26.1)	
**Gender**						
Male	21 (42.0)	29 (58.0)	0.201	143 (72.2)	55 (27.8)	0.490
Female	6 (24.0)	19 (76.0)		78 (76.5)	24 (23.5)	
**Tumor size**						
≤7 cm	9 (42.9)	12 (57.1)	0.056	169 (72.5)	64 (27.5)	0.436
>7 cm	18 (33.3)	36 (66.7)		52 (77.6)	15 (22.4)	
**pT status**						
pT_1-_ pT_2_	21 (33.9)	41 (66.1)	0.527	180 (73.8)	64 (26.2)	1.000
pT_3_ -pT_4_	6 (46.2)	7 (53.9)		41 (73.2)	11 (26.8)	
**pN status**						
pN_0_	23 (32.4)	48 (67.6)	0.031	201 (73.3)	73 (26.7)	0.042
pN_1_ –pN_3_	4 (100.0)	0 (0.0)		20 (90.9)	2 (9.1)	
**TNM stage**						
I-II	17 (29.8)	40 (70.2)	0.056	164 (75.9)	52 (24.1)	0.288
III-IV	10 (55.6)	8 (44.4)		35 (68.6)	16 (31.4)	

^*^ Two sided Fisher’s exact tests.

Some cases were not available for the information in the validation cohort.

### XRCC1 serves as a potential prognostic indicator for ccRCC

To further study whether the reduced expression XRCC1 in ccRCC patients correlates with the prognosis of ccRCC patients, Kaplan-Meier survival curves were constructed by using the 5-year overall and disease-specific cumulative survival rates (n=241, follow-up time of 60 months). Our results revealed that low XRCC1 expression was correlated with both a worse overall and disease-specific survival in ccRCC patients (*P*=0.046 and *P*=0.035, respectively, log-rank test; Figure 1C and 1D).

Furthermore, we examined whether the expression of XRCC1 was an independent prognostic factor for ccRCC patients. We analyzed XRCC1 expression, age, tumor diameter, pT status and TNM stage by univariate and multivariate Cox regression analysis. Our univariate analysis (Table 2) showed that XRCC1, tumor diameter, pT status and TNM stage were correlated with the overall and disease-specific survival of ccRCC patients. In the multivariate analysis (Table 3), only TNM stage remained a significant independent prognosis factor of decreased survival in patients. Taken together, XRCC1 may serve as a predictive biomarker but not as an independent prognostic factor in ccRCC.

**Table 2:** Univariate Cox regression analysis of XRCC1 expression and clinicopathological variables predicting the survival of renal cancer patients

Variables	Overall survival		Disease specific survival	
	HR (95%CI)	*P*	HR (95%CI)	*P*
XRCC1 expression (Low *vs.* High)	0.71(0.50–0.99)	0.049	0.67 (0.46–0.97)	0.038
Age (≤56 *vs.* >56)	1.08 (0.81–1.43)	0.608	1.04 (0.77–1.41)	0.798
Tumor diameter (≤7 cm *vs.* >7 cm)	1.62 (1.14–2.30)	0.007	1.44 (0.96–2.17)	0.008
pT status (pT1/pT2 *vs.* pT3/pT4)	1.52 (1.07–2.15)	0.019	1.34 (0.90–2.00)	0.014
TNM stage (I–II *vs.* III–IV)	1.67 (1.17–2.38)	0.005	1.40 (0.94–2.10)	0.009

Abbreviations: HR: hazard ratio; CI: confidence interval.

**Table 3:** Multivariate Cox regression analysis models assessing the effects of covariates on OS and DSS in CRC patients

Variables	Overall survival		Disease specific survival	
	HR (95%CI)	*P*	HR (95%CI)	*P*
XRCC1 expression (Low *vs.* High)	0.73 (0.50–1.06)	0.101	0.70 (0.47–1.04)	0.079
Age (≤56 *vs.* >56)	1.00 (0.75–1.35)	0.964	0.99 (0.72–1.35)	0.951
Tumor diameter (≤7 cm *vs.* >7 cm)	1.29 (0.88–1.91)	0.196	1.25 (0.81–1.92)	0.315
TNM stage (I–II *vs.* III–IV)	1.69 (1.17–2.44)	0.005	1.44 (0.95–2.17)	0.038

Abbreviations: HR: hazard ratio; CI: confidence interval.

### Silencing XRCC1 in ccRCC cell lines has no effect on cell proliferation

**Figure 2:**
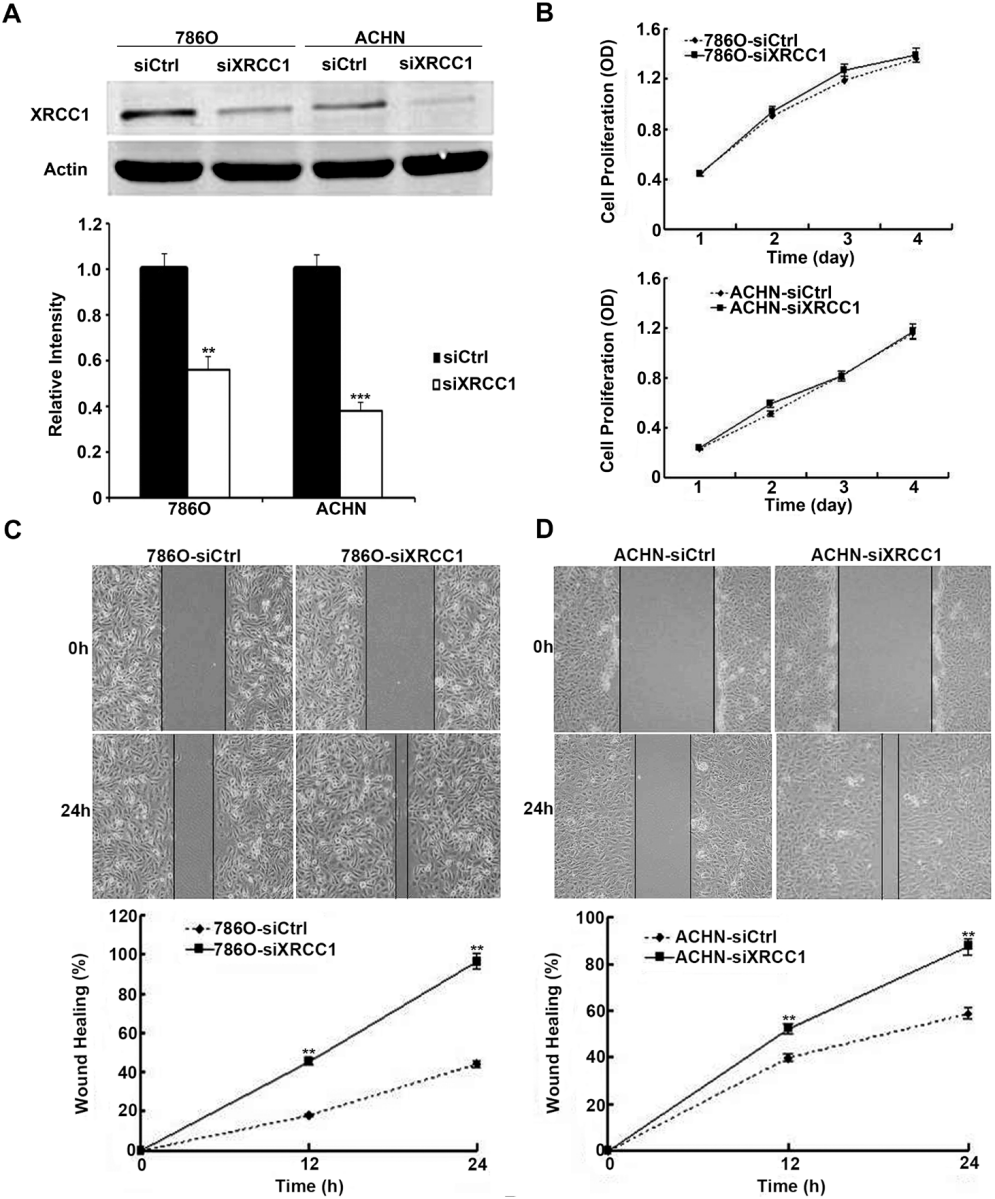
XRCC1 inhibits wound healing of ccRCC cells **(A)** The expressions of XRCC1 were significantly suppressed by XRCC1 siRNA in both 786-O and ACHN cell lines. **(B)** Silencing of XRCC1 had no effects on cell proliferation in both 786-O and ACHN cell lines. **(C)** XRCC1 suppressed wound healing of 786-O cell line. **(D)** XRCC1 suppressed wound healing of ACHN cell line.

Because low XRCC1 expression was associated with poor prognosis in ccRCC patients, XRCC1 may play important roles in the progression of ccRCC. First, we transiently transfected XRCC1 siRNA and control siRNA into 786-O and ACHN cells. Forty-eight hours after transfection, XRCC1 protein expression was significantly decreased in both cell lines (Figure 2A). Then, we investigated the involvement of XRCC1 in ccRCC cell proliferation. In the CCK-8 cell proliferation assay, we found that there was no effect of XRCC1 on ccRCC cell proliferation after XRCC1 silencing in both 786-O and ACHN cells (Figure 2B).

### XRCC1 suppresses the migration and invasion of human ccRCC cells *in vitro*

**Figure 3:**
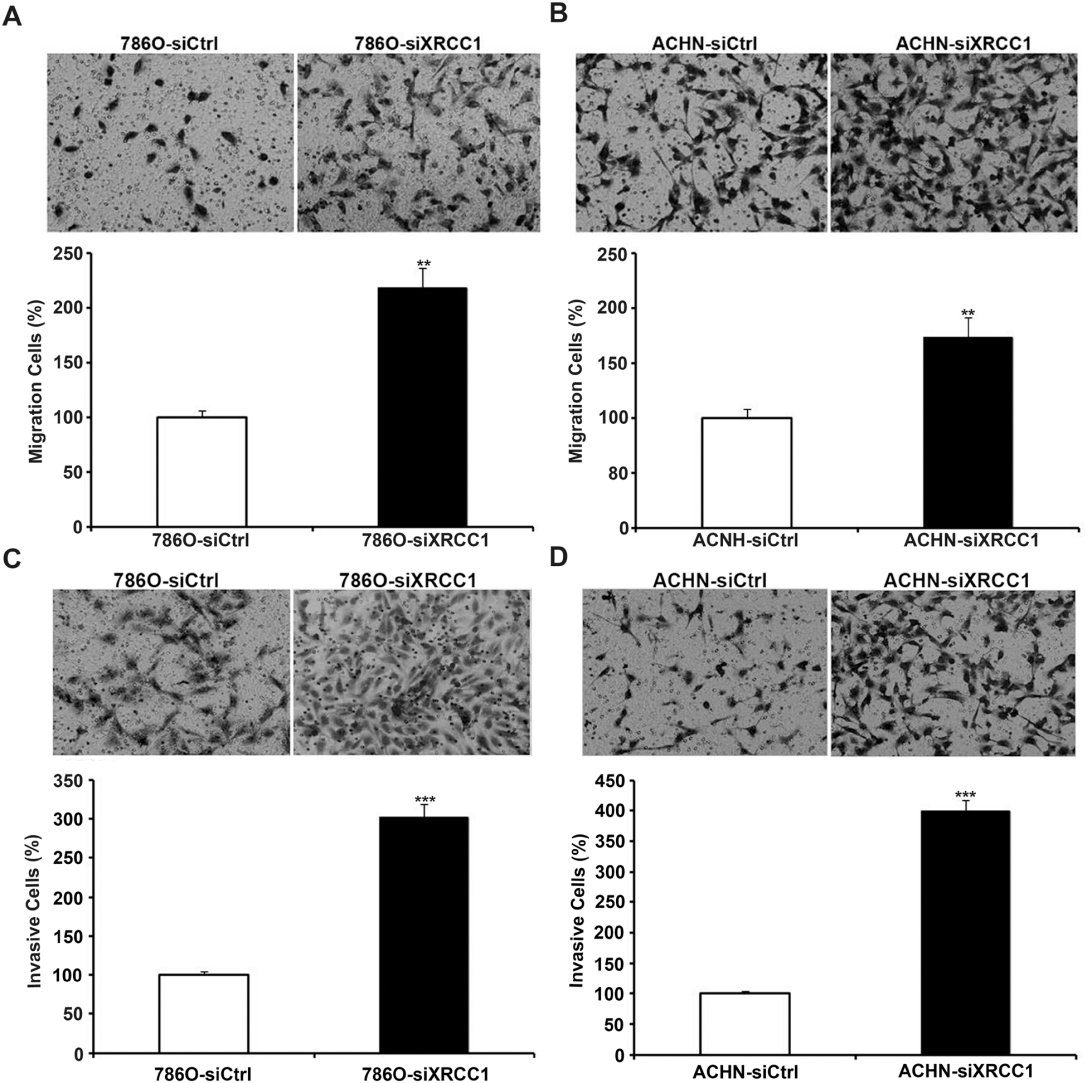
XRCC1 inhibits migration and invasion of ccRCC cells The migration and invasion of XRCC1 knockdown ccRCC cells and controls. **(A, B)** XRCC1 knockdown inhibited migration of 786-O and ACHN cells. **(C, D)** XRCC1 knockdown inhibited invasion of 786-O and ACHN cells.

We continued to examine the effects of XRCC1 on the migration of ccRCC cells through the wound healing and transwell assays. By detecting the width of the scratches in the wound healing assay at 0 h and 24 h (Figure 2C, 2D), we found that the speed of wound healing in 786-O and ACHN cells was faster than that in the control groups. In the transwell filter assay, we found that XRCC1 knockdown significantly enhanced the ability of ccRCC cells to migrate through transwell filter inserts (Figure 3A, 3B). We further examined the effects of XRCC1 on the invasion ability of ccRCC cells. In the cell invasion assay, we got a similar conclusion: XRCC1 knockdown significantly enhanced the ability of ccRCC cells to invade through the transwell filter inserts (Figure 3C, 3D).

### XRCC1 inhibits the migration and invasion of ccRCC cells by suppressing the expression and activity of MMP-2 and MMP-9

**Figure 4:**
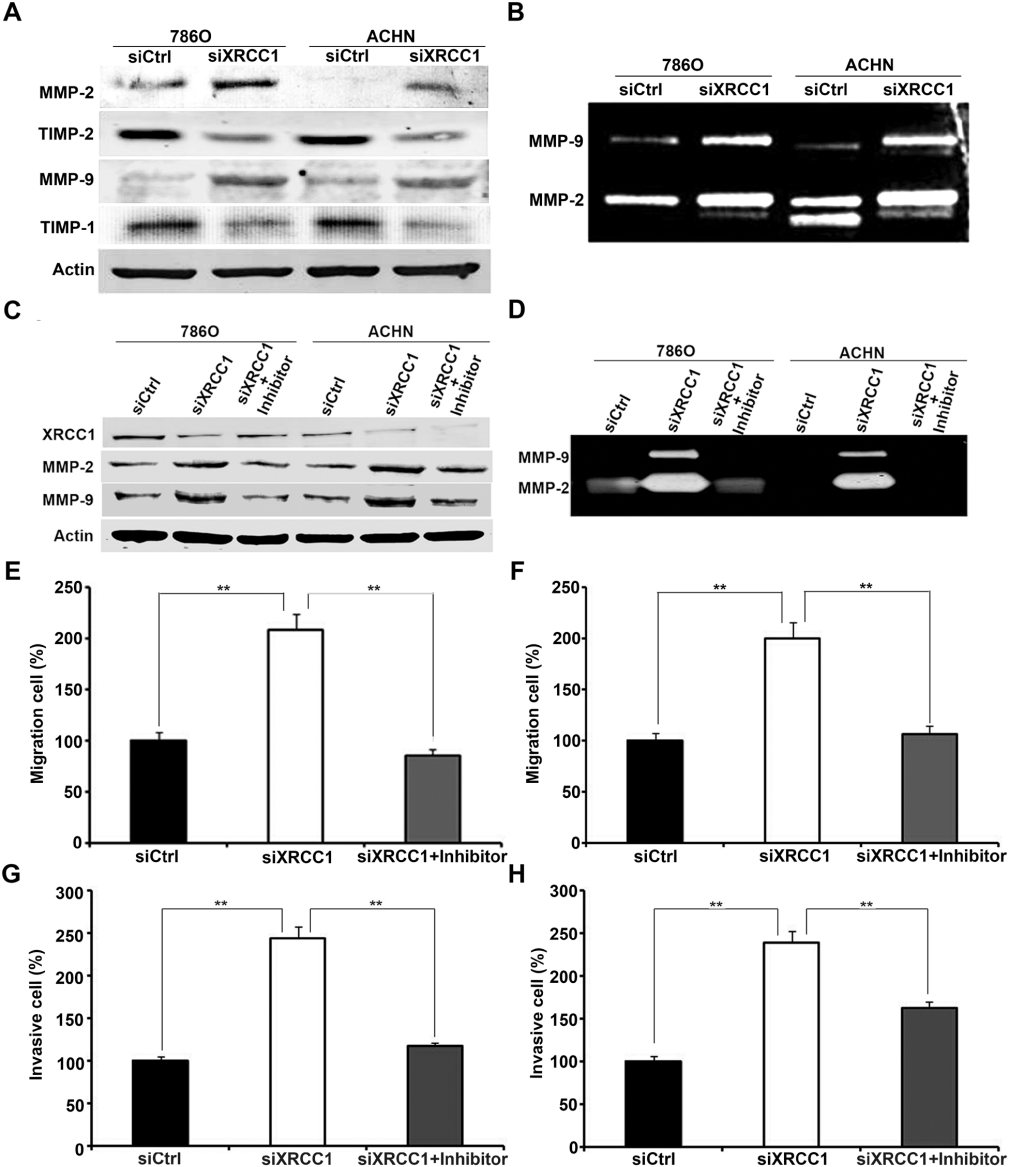
XRCC1 inhibits migration and invasion of ccRCC cells through regulating expressions and activities of MMP-2 and MMP-9 **(A)** The expressions of MMP-2 and MMP-9 were up-regulated dependently of TIMP-2 and TIMP-1 in XRCC1 knockdown in both 786-O and ACHN cell lines detected by Western blot. **(B)** The activities of MMP-2 and MMP-9 were both significantly increased after XRCC1 knockdown in both 786-O and ACHN cell lines detected by Gelatin zymography analysis. **(C)** Western blot results of XRCC1, MMP-2 and MMP-9 from ccRCC cells transfected with the control siRNA, XRCC1 siRNA or co-treated with inhibitor of MMP-2 and MMP-9. **(D)** Gelatin zymography analysis of the activities of MMP-2 and MMP-9 from ccRCC cells transfected with the control siRNA, XRCC1 siRNA or co-treated with inhibitor of MMP-2 and MMP-9. **(E** and **G)** The enhancement of migration and invasion regulated by XRCC1 knockdown in 786-O was blocked by inhibitor of MMP-2 and MMP-9. **(F** and **H)** The enhancement of migration and invasion regulated by XRCC1 knockdown in ACHN was blocked by inhibitor of MMP-2 and MMP-9.

The matrix metalloproteinase (MMP) family has the ability to degrade the extracellular matrix (ECM) during the early stages of many malignant tumors, which plays an important role in the invasion and metastasis of tumors [9]. To investigate the mechanisms by which XRCC1 regulates the migration and invasion of ccRCC cells, we detected MMP protein expression and activity levels in 786-O and ACHN cells by western blot and gelatin zymography assays. Our data showed that MMP-2 and MMP-9 expression and activity were negatively regulated by XRCC1 expression in ccRCC cells (Figure 4A, 4B). Therefore, we supposed that XRCC1 could suppress the migration and invasion of ccRCC cells by regulating MMP-2 and MMP-9. To further validate our hypothesis, we added a selective inhibitor of MMP-2/MMP-9 (sc-311429, Santa Cruz). At the same time, XRCC1 siRNA was transfected into ccRCC cells. As expected, the up-regulation of both MMP-2 and MMP-9 expression and activity was blocked by a selective inhibitor of MMP-2 and MMP-9 (Figure 4C, 4D). We further validated these phenomena by migration and invasion assays. Migration and invasion ability were enhanced by inhibiting XRCC1 in ccRCC cells; however, this effect was blocked by the selective inhibitor of MMP-2 and MMP-9 (Figure 4E-4H). Above all, our results confirmed that XRCC1 expression inhibited the migration and invasion of ccRCC cells by suppressing MMP-2 and MMP-9 expression and activity.

It is well known that tissue inhibitors of matrix metalloproteinases (TIMPs) have the ability to inhibit the catalytic activities of MMPs. The imbalance between MMPs and TIMPs is responsible for cancer metastasis. TIMP-1 and TIMP-2 are inhibitors of MMP-9 and MMP-2, respectively. To understand whether XRCC1 regulates the expression and activity of MMP-2 and MMP-9 by TIMP-2 and TIMP-1, we further detected the expression level of TIMP proteins. Our results showed that the expression of both TIMP-2 and TIMP-1 decreased when MMP-2 and MMP-9 were up-regulated following XRCC1 knockdown (Figure 4A), which indicated that XRCC1 can regulate MMP-2 and MMP-9 expression through TIMP-2 and TIMP-1 in ccRCC.

## DISCUSSION

In 1990, XRCC1 was the first human gene involved in DNA single-strand breaks to be cloned [10]. Many studies have indicated that XRCC1 expression is up-regulated in tumor progression and might be a risk factor for tumor progression [8, 11]. However, a recent publication showed a lack of correlation between XRCC1 gene polymorphisms and colorectal cancer susceptibility in a Malaysian cohort [12]. In addition, it has been verified that XRCC1 has a low expression level in many kinds of cancers, and XRCC1 has been proposed to serve as an anti-cancer marker for diagnosis. Loss of XRCC1 expression was correlated with the progression of melanoma from AJCC stage II to stage III and with worse overall and disease-specific 5-year and 10-year survival in 119 melanoma patients. Furthermore, XRCC1 expression has also been depicted as having an inhibitory effect on melanoma cell invasion and migration [13]. These results suggest that XRCC1 may play multifaceted roles in tumor development, progression and metastasis.

In our present study, we used two independent ccRCC cohorts including patients from the Shanghai and Xuzhou area of China. The advantage of this design is that the results of the two separate cohorts can be mutually validated. We found that the expression of XRCC1 was downregulated in all ccRCC specimens and that the low expression level in ccRCC was strongly related to lymph node metastasis, indicating that XRCC1 might play anti-cancer roles in ccRCC. Furthermore, low XRCC1 expression correlated with a worse overall and disease-specific survival in ccRCC patients. However, XRCC1 expression was not associated with worse survival in multivariable analyses, which suggested that XRCC1 cannot be used as an independent prognostic factor.

A recent publication studied the impact of plasmacytoid variant histology on the survival of patients with urothelial carcinoma of the bladder after radical cystectomy; a large cohort of patients indicated that the plasmacytoid variant was associated with adverse pathologic features but was not associated with worse overall mortality in multivariable analyses [14]. Our results were similar to these. The prognosis of cancer patients is the result of comprehensive factors; the impact of other factors cannot be determined in this study. Despite this limitation, our large retrospective series highlighted the lymph node metastasis characteristics of ccRCC with downregulated XRCC1 expression.

A recent publication showed that vascular invasion was significantly more frequent in patients with biliary tract cancers with a low expression of XRCC1 [15]. Metastasis of ccRCC cells remains the major cause of mortality in patients with ccRCC. Therefore, we further examined the effects of XRCC1 expression on the migration and invasion of ccRCC cells. Our data demonstrated that XRCC1 had the ability to inhibit ccRCC cell migration and invasion. These results prompted us to carry out a series of *in vitro* experiments to explore the potential mechanisms of this inhibitory activity.

Tumor metastasis has multistage processes consisting of tumor cell proliferation, migration, invasion, intravasation, survival in blood circulation, extravasation, arrest at distant organ sites, micro metastasis formation and metastatic colonization, et al. Our studies have found that silencing XRCC1 expression had no effect on cell proliferation ability in ccRCC. The results indicated that XRCC1 does not regulate the metastasis of ccRCC through cellular proliferation. MMPs, a family of zinc-dependent endopeptidases, are involved in the degradation of extracellular matrix (ECM), contributing to cell invasion and metastasis, angiogenesis and tumorigenesis. MMP-2 and MMP-9 act as type IV collagenases and can specifically degrade the prominent component of the basement membrane [16].

The expression of MMP-9 and MMP-2 is considered an important sign of tumor invasion due to the critical roles the basement membrane plays in the process of tumor invasion. Our data demonstrated that the downregulation of XRCC1 expression by siRNA transfection significantly enhanced the expression and activity of MMP-2 and MMP-9. In addition, the up-regulation of MMP-2 and MMP-9 expression and activity were both blocked by a selective inhibitor of MMP-2 and MMP-9.

The expression and activity of MMPs are influenced by numerous factors, e.g., hormones, cytokines, and growth factors, such as epidermal growth factor (EGF) and transforming growth factor-beta (TGF-β) [17]. The precise control of MMP activity occurs through TIMPs [18]. TIMPs are considered endogenous inhibitors of MMPs, modulating MMP-mediated ECM degradation. The catalytic activities of MMP-2 and MMP-9 are controlled by interacting with TIMP-2 and TIMP-1, respectively. Our results showed that the downregulation of XRCC1 by siRNA significantly enhanced the expression of MMP-2 and MMP-9, while the expression of TIMP-2 and TIMP-1 were decreased (Figure 4A). Therefore, we confirmed that XRCC1 inhibited ccRCC migration and invasion by inhibiting MMP-2 and MMP-9 expression through the regulation of TIMP-2 and TIMP-1.

In summary, based on our findings, we conclude that the loss of XRCC1 expression was significantly correlated with the progression of ccRCC and was related to worse ccRCC patient survival but was not an independent predictor of survival in ccRCC patients. Furthermore, we have confirmed that XRCC1 can suppress ccRCC migration and invasion by inhibiting MMP-2 and MMP-9 expression through the regulation of TIMP-2 and TIMP-1.

## MATERIALS AND METHODS

### Patients and specimens

Two independent ccRCC tissue microarray (TMA) cohorts were utilized in our study. The training cohort TMA was purchased from Shanghai Xinchao Biotechnology (Shanghai, China). The ccRCC tissues and paired non-cancerous tissues were obtained from 75 patients who underwent radical nephrectomy between 2006 and 2008. The diameter of every array dot was 1.5 mm, and each dot represented a tissue from one individual specimen.

The validation cohort TMA was constructed at the National Engineering Center for Biochip (Shanghai, China) by a contract service and consisted of 300 surgical cases and 35 cases of normal renal tissue. The surgical specimens were collected from patients with ccRCC who underwent radical nephrectomy without prior treatment at the Affiliated Hospital of Xuzhou Medical University between 2005 and 2008. All the tissue specimens for our present experiment were obtained with informed patient consent and were approved by the Review Board of the Affiliated Hospital of Xuzhou Medical University. The clinicopathological information collected from the ccRCC patients included age at diagnosis, gender, depth of tumor invasion, tumor diameter, lymph node metastasis and TNM stages. Of the 300 patients, the 243 patients living in the Xuzhou area were followed until their death or until their most recent contact.

### Immunohistochemistry

Immunohistochemistry was performed as described before [19]. A mouse anti-XRCC1 monoclonal antibody (ab1838, Abcam, US) was used at a dilution of 1:200. An HRP-conjugated secondary antibody (12127A07, Beijing Sequoia Jinqiao Biological Technology Co., Ltd.) was used. The specific target(s) was visualized with a 3,3’-diaminobenzidine (DAB) detection kit (Beijing Sequoia Jinqiao Biological Technology Co., Ltd.) and counterstained with hematoxylin.

### Evaluation of immunostaining

Positive XRCC1 protein staining appeared brown in the nucleus with or without staining in the cytoplasm. We graded the positive staining according to both the percentage of cells stained and the stain intensity. All slides were independently examined by two pathologists. The intensity of the XRCC1 staining was scored 0 to 3 (0=negative, 1=moderate; 3=strong). The percentage of positively-stained cells was scored according to 4 levels: 1 (0-25% of cells positively stained), 2 (26-50%), 3 (51-75%) and 4 (76-100%). In cases of discrepancy between the two scores, the 2 individual tissue scores were averaged for the final score. The level of protein staining was evaluated by the immunoreactive score (IRS), which was calculated by multiplying the percentage score by the intensity score of the positive cells. Based on the IRS, the staining pattern of the specimens was defined as negative (IRS: 0), weak (IRS: 1-3), moderate (IRS: 4-6), or strong (IRS: 8-12).

### Cell lines

The human ccRCC cell lines 786-O and ACHN were purchased from the Shanghai Institute of Biochemistry and Cell Biology, Chinese Academy of Science (Shanghai, China). Cells were cultured as described before [20]. 786-O cells were cultured in Roswell Park Memorial Institute 1640 medium (RPMI 1640, Invitrogen, Shanghai, China) supplemented with 10% fetal calf serum (Invitrogen); ACHN cells were cultured in minimum essential medium (MEM, Invitrogen) supplemented with 10% fetal calf serum. These two cell lines were both incubated in a 37°C humidified incubator with 5% CO_2_.

XRCC1 siRNA (ATCTTCTCAAGGCAGACAC) and nonspecific control siRNA were purchased from GenePharma (Shanghai, China). XRCC1 siRNA and nonspecific control siRNA were both transfected into 786-O and ACHN cells with siLentFect Lipid Reagent (Bio-Rad, Hercules, CA, USA) according to the manufacturer’s instructions.

### Cell proliferation assay

Cell proliferation was analyzed by using a WST-8 Cell Counting Kit-8 (Beyotime, Nantong, China). We transiently transfected 786-O and ACHN cells with XRCC1 siRNA or control siRNA. 786-O and ACHN transfected cells were plated into 96-well plates at a density of 3×10^3^ and 2×10^3^ cells/well, respectively, and cells were incubated at 37°C for 24, 48, 72 or 96 h. CCK-8 solution (10 μl) was added to each well after 24, 48, 72 or 96 h, and the cultures were incubated at 37°C. Absorbance at 450 nm was measured on an ELX-800 spectrometer reader (Bio-Tek Instruments, Winooski, USA) after 1 h of incubation.

### Wound healing assay

Wound healing assays were used to detect cell migration ability. After transfecting 786-O and ACHN cells with XRCC1 siRNA and control siRNA, cells were grown to confluency, a wound line was made by scraping a closed Pasteur pipette tip across the confluent cell layer. Then, cells were washed three times with PBS to remove detached cells and debris. The size of the wound was observed and measured after 24 hours.

### Cell migration and invasion assay

Cell migration and invasion assays were performed by using modified two chamber plates with a pore size of 8 μm. The transwell filter was inserted with or without Matrigel (BD Biosciences) coating for invasion or migration assays, respectively. The detailed conditions have been described previously [19].

### Gelatin zymography

Gelatin zymography was performed as described before [19]. It was used to detect the activities of MMP-2 and MMP-9. Cells (2.5 × 10^6^) were seeded in 100-mm plates and cultured for 24 h. Then, proteins in the conditioned medium were collected and concentrated using Amicon Ultra-4-30 k centrifugal filters (Millipore, Billerica, MA, USA) at 7500 g for 20 min at 4°C. We loaded twenty micrograms of protein in non-denaturing conditions into a 10% polyacrylamide gel containing 0.1% gelatin (Sigma, St. Louis, MO, USA). After electrophoresis, gels were washed in 2.5% Triton X-100 for 30 minutes with a single change of the detergent solution. Then, gels were incubated for 42 h at 37°C in incubation buffer (50 mM Tris-HCl (pH 8.8), 5 Mm CaCl_2_, 1 μM ZnCl_2_, and 0.02% NaN_3_), stained with 0.1% Coomassie brilliant blue R-250 (Sigma) for at least 4 h, and destained in 10% acetic acid and 45% methanol for 2 h. Gelatinolytic activity was shown as a clear area in the gel. Then, gels were photographed and quantitatively measured by scanning densitometry.

### Antibodies and western blot (WB)

Antibodies against the following proteins were used: XRCC1 (1:200); MMP-2 (1:100, Cell Signaling Technology, Beverly, MA, USA); MMP-9 (1: 200, Cell Signaling Technology); TIMP-1 (1: 200, Santa Cruz); TIMP-2 (1: 200, Santa Cruz); and β-actin (1: 1000, Cell Signaling Technology). Infrared IRDye-labeled secondary antibody (1: 10000, LI-COR, Lincoln, NE, USA) was applied to the blot for 1 hour at room temperature. The signals were detected by an Odyssey Infrared Imaging system (LI-COR).

western blot analysis was performed as described previously [19]. Cells were harvested and washed twice with PBS; then, whole-cell proteins were extracted as described previously. Protein concentration was determined by a protein assay (Bio-Rad). All protein samples were denatured, electrophoresed in SDS/polyacrylamide gels and transferred into polyvinylidene difluoride membranes (Millipore).

### Statistical analysis

Data are expressed as the means ± SDs. Statistical analysis for the TMA was performed by SPSS 20 (SPSS, Inc, Chicago, IL). The association between the staining of XRCC1 and the clinicopathological parameters of ccRCC patients, including age, gender, tumor size, depth of tumor invasion, lymph node metastasis and TNM stage, was evaluated by the two-sided Fisher’s exact test. The difference between the XRCC1 IRSs in tumors and in paired adjacent normal renal tissues was assessed by the Wilcoxon test (grouped). The correlation between XRCC1 expression and patient survival was assessed by Kaplan-Meier and log-rank tests. Univariate and multivariate analysis was analyzed by a Cox regression model. Two-factor analysis of variance and Dunnett’s t-test were used to assess differences within treatment groups. Differences were considered significant when *P*< 0.05.
